# Popliteal Entrapment in a 16‐Year‐Old Soccer Player Treated via Detachment and Repositioning of the Medial Head of the Gastrocnemius

**DOI:** 10.1155/cro/6625479

**Published:** 2025-12-19

**Authors:** Thomas Bane, Nadia Nawabi, Kirk Hance, Jacob Brubacher

**Affiliations:** ^1^ Department of Orthopedic Surgery, The University of Kansas Medical Center, Kansas City, Kansas, USA, kumc.edu; ^2^ Department of Vascular Surgery, The University of Kansas Medical Center, Kansas City, Kansas, USA, kumc.edu

## Abstract

**Background:**

Popliteal artery entrapment syndrome (PAES) is a frequently underdiagnosed disease in young patients due to its low incidence and nonspecific symptoms. The disease is classified broadly into two categories: anatomic entrapment or functional entrapment. Prompt diagnosis is crucial due to the potentially limb‐threatening nature of this condition should critical limb ischemia develop.

**Case:**

A 16‐year‐old male soccer player with no past medical history who had 1 week of acute onset right lower extremity calf pain and foot numbness due to right proximal popliteal and tibial artery occlusion in the setting of Type 1 popliteal artery entrapment. Treatment initially included thrombolysis but was complicated by development of compartment syndrome requiring emergent four‐compartment fasciotomy. He subsequently underwent thrombectomy of his popliteal, anterior, and posterior tibial arteries before definitive popliteal artery decompression with medial gastrocnemius recession and repositioning.

**Discussion:**

PAES is a rare disease that can lead to devastating complications such as critical limb ischemia as seen in this patient. It is of paramount importance to identify this disease early and implement appropriate treatment. Debate remains as to the necessity of bypass or vascular repair in patients with Type 1 PAES. This patient did not undergo popliteal artery bypass and has been doing well postoperatively thus far.

## 1. Background

Popliteal artery entrapment syndrome (PAES) is a rare disease with a 15:1 male‐to‐female ratio [1] and an approximate incidence ranging from 0.6% to 3.5% [2]. Once the disease has been identified, it is important to assess the contralateral leg as bilateral disease has been reported in upwards of 67%–81% of cases [3, 4]. PAES is broadly classified into either anatomic entrapment when aberrant muscular or tendinous anatomy leads to compression and injury to the neurovascular structures, or functional entrapment (Type VI) when there is no anomalous popliteal fossa anatomy, but the neurovascular structures become compressed during patient movement. Further subtypes of anatomic entrapment have been described depending on the anatomy of the popliteal entrapment. Type I refers to when the popliteal artery courses medial to the medial head of the gastrocnemius (MHG). Type II refers to a lateral origin of the MHG which causes the popliteal artery to pass medial. Type III refers to either an accessory muscle or abnormal fibrous band that arises from the medial or lateral femoral condyle and wraps circumferentially around the popliteal artery. Type IV refers to a popliteal artery that lies deeper within the fossa than normal, causing it to be compressed by either the popliteus muscle or fibrous bands. Type V refers to when both the popliteal artery and vein are entrapped by any of the aforementioned anatomic variations. Types 2 (25%) and 3 (30%) are the most common [5, 6]. It is difficult to diagnose popliteal artery entrapment due to its nonspecific symptoms that can mimic exertional compartment syndrome or other similar conditions [7, 8]. Diagnosis typically includes ankle–brachial indices of bilateral lower extremities, duplex ultrasound imaging, and MRA versus CTA to assess vascular anatomy for surgical planning [9]. Treatment varies depending on the type of entrapment, presence of vascular injury, and chronicity of the disease [4, 10].

## 2. Case Report

The patient in question and his mother provided consent for the documentation of their medical diagnosis and treatment for the education of future clinicians as a case report. The patient is a 16‐year‐old male who reports to the vascular surgery clinic with a chief complaint of right leg pain that worsens with walking as well as pallor to his right foot. He has no medical history and this pain started suddenly 1 week prior to presentation. There is no pain at rest. On exam, the patient does not have a palpable pulse in the right dorsalis pedis or posterior tibial arteries. ABI′s on the right are 0.7 compared to 1.15 on the left. An MR angiogram obtained at an outside facility demonstrated a thrombus that extended from the proximal popliteal artery to the distal superficial femoral artery. The outside radiology report did not identify any aberrant popliteal artery anatomy; however, on independent review, our vascular surgery team identified the popliteal artery traveling medial to the MHG at the popliteal fossa. He was subsequently admitted to the hospital for a hypercoagulable panel, an embolic source work‐up, and initiation of anticoagulation. Work‐up did not identify any evidence of hypercoagulability or embolic source of the occlusion.

**Figure 1:**
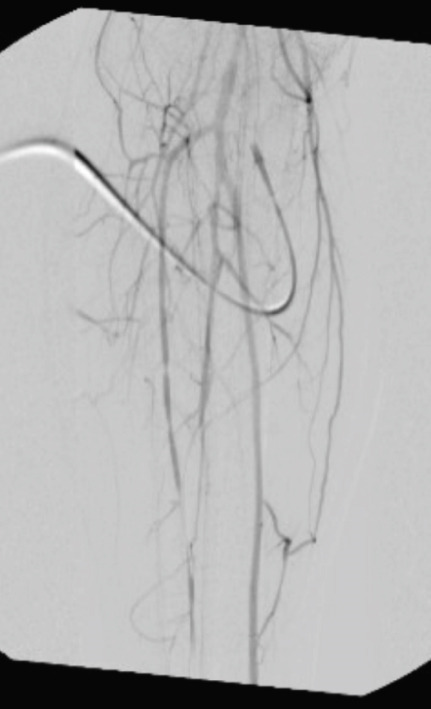
Intraoperative angiogram at the popliteal fossa.

**Figure 2:**
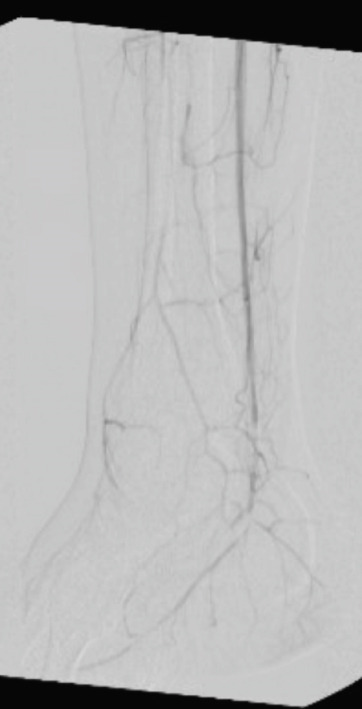
Intraoperative angiogram at the level of the ankle.

**Figure 3:**
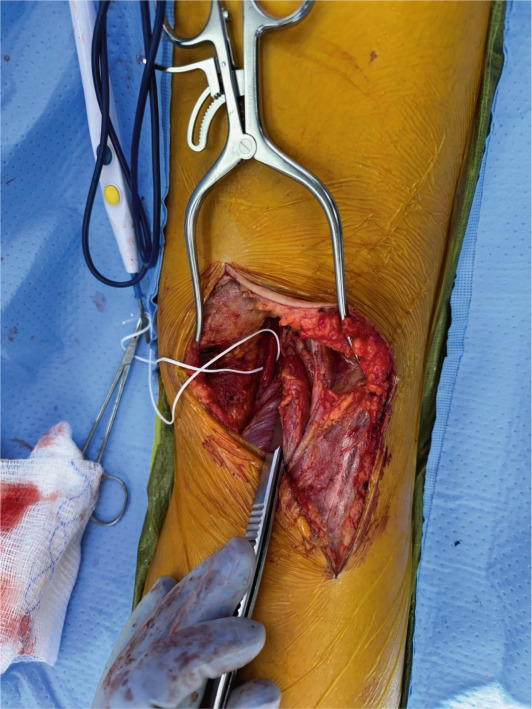
Vessel loop around popliteal artery as it traverses medial to the medial head of the gastrocnemius. The left side of the image is the patient′s medial side.

**Figure 4:**
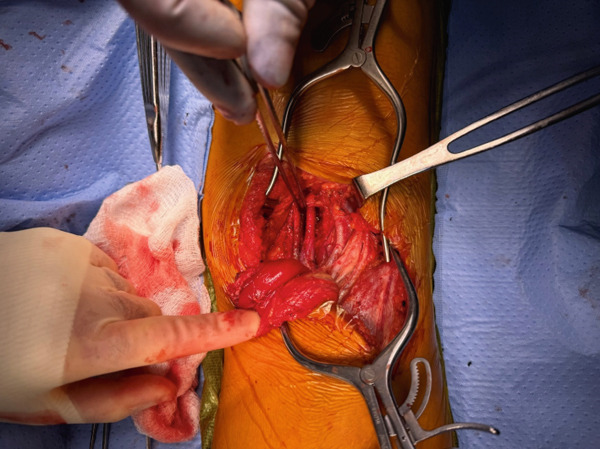
Origin of the medial head of the gastrocnemius transected and the popliteal artery is shown being mobilized to its usual anatomic position. Left side of the image is patient′s medial side.

The patient initially underwent percutaneous thrombolysis of the area of occlusion with the interventional radiology team. This was complicated by the development of compartment syndrome on hospital Day 3 requiring emergent four‐compartment fasciotomy and removal of the lysis catheter. The next day, the patient was taken back to the operating room for a right popliteal artery thrombectomy using the medial fasciotomy incision, a right posterior tibial artery thrombectomy via an incision just posterior to the medial malleolus, and a right anterior tibial thrombectomy through the anterior compartment fasciotomy incision. Intraoperative angiogram confirmed inline flow through the anterior and posterior tibial arteries, with few segmental areas of narrowing that were thought to be due to spasm. No evidence of intimal defect was identified during final intraoperative angiogram as shown in Figures 1 and 2. Doppler assessment showed biphasic flow was present in both posterior tibial and anterior tibial arteries after the procedure. On hospital Day 5, the patient returned to the operating room for release of the right popliteal artery entrapment. A standard posterior approach to the knee was performed allowing for easy identification of the popliteal artery traversing medial to the medial head of the gastrocnemius as shown in Figure 3. The origin of the medial head of the gastrocnemius from the distal femur was released, and the popliteal artery was mobilized into a centralized location as shown in Figure 4. The medial head of the gastrocnemius was repositioned back to the origin site using two fiber tack suture anchors placed at the footprint of the medial head of the gastrocnemius. The muscle was secured to the suture in a locking Krakow fashion and the repair was judged to be stout. A good pulse in the popliteal artery was palpated after re‐attachment of the medial head of the gastrocnemius and there was no evidence for compression of the artery. After 3 additional days for adequate pain control and plan for chronic anticoagulation, the patient was discharged from the hospital. Of note, plastic surgery was involved in placing a split‐thickness skin graft over the lateral fasciotomy site. The graft epithelialized without complication as of 8‐week postoperative appointment with plastic surgery.

**Figure 5:**
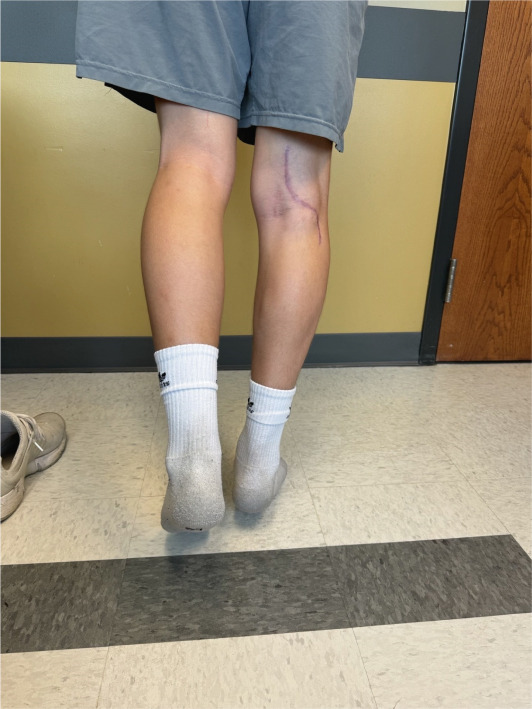
Then, 8 weeks postoperatively, patient is able to perform straight leg raise with well healed incision.

The patient was discharged from the hospital 3 days after medial head of the gastrocnemius release and re‐insertion. He had patent flow in his right lower extremity with biphasic Doppler signals at his DP, AT, and PT arteries. He was allowed to bear weight on the operative side but was instructed to limit active knee and ankle range of motion. He was allowed to do passive range of motion at the knee and ankle as tolerated. At the first postoperative visit 2 weeks later, the patient′s AT signal was monophasic and weaker than at time of discharge; however, his PT and plantar vessel doppler signals were unchanged from discharge. The orthopedic team assessed the patient as well at that time and noticed a significant 45° flexion contracture of the knee. The patient was cleared for all activities and was seen again 8 weeks later by the vascular and orthopedic surgery teams. At 8 weeks postoperatively, the patient′s ABI in his right leg was 0.79. His symptoms were resolved at that time and he remains asymptomatic in his left leg. After a long discussion with family and using shared decision‐making, we elected to not pursue further advanced imaging on the patient′s left leg since he is not having any symptoms. He had returned to full activity and resumed running without difficulty. The patient′s knee flexion contracture had entirely resolved, and he was able to actively contract both the medial and lateral heads of the gastrocnemius to perform a single leg raise as shown in Figure 5. The vascular surgery team will plan to repeat ABI′s every 3 months for the first year postoperatively, then every 6 months for the second year postoperatively, and then yearly starting the third year postoperatively.

## 3. Discussion

This case report describes the initial assessment and ultimate surgical management of a patient with Type 1 popliteal entrapment syndrome. Various classification systems have been created to describe PAES and are either based on anatomic or functional entrapment [5]. When focusing on anatomic entrapment, the pathoanatomy can either be related to the course of the popliteal artery, atypical muscular insertion, or some combination of the two [11]. This patient′s pathoanatomy was due to his popliteal artery traversing medial to the medial head of the gastrocnemius.

Our patient, being a young male athlete without prior atherosclerotic risk factors, fits the stereotypical risk factors and patient characteristics for PAES [1]. His history, however, was atypical for popliteal entrapment syndrome. Most commonly, patients describe intermittent and insidious claudication symptoms rather than an acute onset. In the literature, close to 11% of patients report an acute onset of symptoms in their presenting history [5]. In that study, the authors postulated that a focal thrombus in the popliteal artery can lead to distal emboli and subsequent acute limb ischemia [5]. Igari et al. corroborate this finding in their study of 29 limbs that underwent surgical management for PAES. Seven limbs demonstrated radiographically evident distal emboli, with three out of 29 (10.3%) patients reporting sudden onset of symptoms [12]. Our patient presented with an acute onset of symptoms and was ultimately found to have a thrombus in his popliteal artery with distal emboli to his peroneal artery requiring thrombectomy.

Evaluation of the contralateral leg for disease is crucial in this condition as the rate in the literature shows that entrapment occurs bilaterally in 20%–81% of patients [3, 4, 12]. Our patient was asymptomatic in his left leg and had normal ABI′s of 1.15. Nevertheless, a high degree of clinical suspicion should be maintained when evaluating the “well‐leg” of a patient with newly diagnosed PAES.

The goal of surgical management for PAES is to ameliorate patient symptoms by releasing extrinsic compression around the popliteal artery and maintaining patient flow either through or around the popliteal artery. Releasing extrinsic compression around the popliteal artery depends on the pathoanatomy of the patient′s disease. Sadri et al. describe an example of this for a patient with Type 3 PAES [13]. The authors excised an accessory slip of the gastrocnemius that arose from the medial head of the gastrocnemius and compressed the popliteal artery [13]. Similarly, the cohort studied by Igari et al. underwent resection of musculotendinous slips to release the popliteal artery [12]. Restoring adequate blood flow to the distal extremity requires close examination of the popliteal artery. There is evidence to support no further intervention upon the popliteal artery other than release of extrinsic compression. In the case report by Sadri et al., the authors describe only releasing the musculotendinous slip of the gastrocnemius that compressed the patient′s popliteal artery. That patient went on to return to normal function at 8 weeks postoperatively [13]. Similarly, in a case series by Levien et al., 66 patients were treated with only musculotendinous resection without bypass of the popliteal artery, and all patients maintained a patent popliteal artery at a median of 3.9 year follow‐up [14]. Fully releasing the extrinsic compression about the popliteal artery portends better outcomes in these patients. Igari et al. describe three failures of primary graft patency, with two of those failures being due to hypertrophy of remnant musculotendinous slips [12]. Thus, for our patient, a complete release of the medial head of the gastrocnemius, transposition of the popliteal artery, and reinsertion of the gastrocnemius into the distal femur was performed to adequately decompress the popliteal artery.

When the popliteal artery requires intervention, options include thrombectomy with patch angioplasty, autogenous vein bypass graft, or segmental arterial resection with graft interposition [14]. When there is popliteal artery stenosis, the complication rate is significantly higher with thrombectomy and vein patching (16.7%) compared to other revascularization procedures (45.5%) [15]. Comparing interposition procedures with bypass surgeries, Kim et al. showed that bypass surgery had a lower patency rate at 5‐year follow‐up than interposition procedures: 30.0%–85.9%, respectively [16]. At the time of definitive surgery, the popliteal artery of our patient appeared patent and undamaged, so the decision was made to not perform any bypass or interposition grafting procedures.

## 4. Conclusion

This is a case of PAES in a 16‐year‐old male athlete who presented with acute onset of right leg claudication symptoms and abnormal ABI′s. Treatment initially included thrombolysis but was complicated by the development of compartment syndrome requiring emergent four‐compartment fasciotomy. He subsequently underwent thrombectomy of his popliteal, anterior, and posterior tibial arteries before definitive popliteal artery decompression with medial gastrocnemius recession and repositioning. No bypass surgery or interposition graft procedure was performed on the popliteal artery. The patient progressed well after surgery with resolution of claudication symptoms and return to normal activities at 8 weeks postoperatively.

## Conflicts of Interest

The authors declare no conflicts of interest.

## Funding

No funding was received for this manuscript.

## Data Availability

The data that support the findings of this study are available on request from the corresponding author. The data are not publicly available due to privacy or ethical restrictions.
